# Nanostructured bulk Si for thermoelectrics synthesized by surface diffusion/sintering doping

**DOI:** 10.1039/c9ra02349f

**Published:** 2019-05-17

**Authors:** Sora-at Tanusilp, Naoki Sadayori, Ken Kurosaki

**Affiliations:** Graduate School of Engineering, Osaka University 2-1 Yamadaoka, Suita Osaka 565-0871 Japan; Nitto Denko Corporation 1-1-2 Shimohozumi, Ibaraki Osaka 567-8680 Japan naoki.sadayori@nitto.com; Research Institute of Nuclear Engineering, University of Fukui 1-3-33 Kanawa-cho, Tsuruga Fukui 914-0055 Japan; JST, PRESTO 4-1-8 Honcho, Kawaguchi Saitama 332-0012 Japan; Institute for Integrated Radiation and Nuclear Science, Kyoto University 2, Asashiro-Nishi, Kumatori-cho, Sennan-gun Osaka 590-0494 Japan kurosaki.ken.6n@kyoto-u.ac.jp

## Abstract

Nanostructured bulk silicon (bulk nano-Si) has attracted attention as an advanced thermoelectric (TE) material due to its abundance and low toxicity. However, oxidization will occur easily when bulk nano-Si is synthesized by a conventional method, which deteriorates the TE performance. Various methods to prevent such oxidation have been proposed but they need specific techniques and are thus expensive. Here, we propose a simple and cost-effective method named Surface Diffusion/Sintering Doping (SDSD) to synthesize bulk nano-Si for TEs. SDSD utilizes Si nanoparticles whose surface is coated with a native thin oxide layer. SDSD is composed of two steps, (1) a molecular precursor containing a doping element is added onto the oxide layer of Si nanoparticles and (2) the nanoparticles are sintered into a bulk state. During sintering, the doping element diffuses through the oxide layer forming conductive paths, which results in a high carrier concentration as well as high mobility. Furthermore, owing to the nanostructures, low lattice thermal conductivity (*κ*_lat_) is also achieved, which is an ideal situation for TEs. In this study, we show that P-doped bulk nano-Si synthesized by SDSD shows good TE performance due to its high carrier concentration, high carrier mobility, and low *κ*_lat_. Since SDSD takes advantage of oxidization, it is cost-effective and suitable for mass production to synthesize bulk nano-Si for TEs.

## Abstract 2

Nanostructured bulk silicon (bulk nano-Si) has attracted attention as an advanced thermoelectric (TE) material due to its abundance and low toxicity.

## Graphical abstract

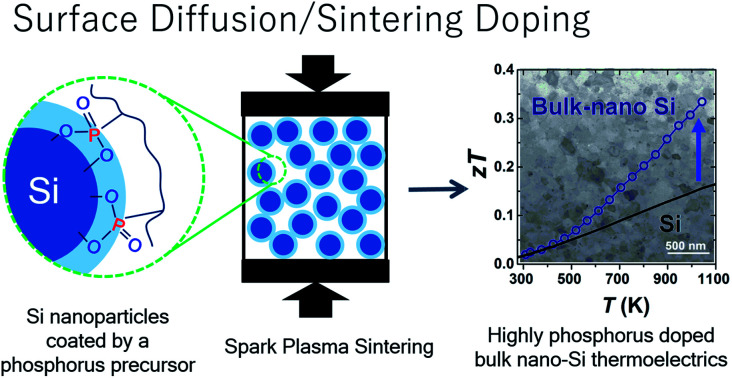

## 1. Introduction

Thermoelectrics (TEs) can convert heat gradients into electricity and *vice versa*, making them important to the future of power generation from waste heat.^1^ The effectiveness of TE materials is quantified by the dimensionless figure of merit (*zT* = *S*^2^*σT*/(*κ*_el_ + *κ*_lat_), where *S*, *σ*, *T*, *κ*_el_, and *κ*_lat_ are the Seebeck coefficient, electrical conductivity, absolute temperature, and electronic and lattice components of the thermal conductivity, respectively).^2^ Current TE materials such as Bi_2_Te_3_ and PbTe contain highly toxic and/or rare elements, which limits their wide utilization in consumer markets. Thus, many wish to develop a high-*zT* material made from inexpensive, non-toxic, and Earth-abundant elements.

Si is a typical representative of such elements. Conventional bulk Si exhibits good electrical properties (*i.e.*, high *S*^2^*σ*), while its *κ*_lat_ is high (>100 W m^−1^ K^−1^), leading to a *zT* of ∼0.01 at room temperature.^3^ An ideal way to enhance *zT* is by scattering phonons without scattering electrons, which leads to decrease in *κ*_lat_ with maintaining high *S*^2^*σ*, results in enhanced *zT*. This situation can be realized by nanostructuring.^4–7^

A traditional method to synthesis nanostructured bulk Si (bulk nano-Si) is consolidating fine nanoparticles of highly doped Si through ball milling (BM) followed by hot pressing or spark plasma sintering (SPS). This method has been demonstrated to synthesis various nanostructured bulk TE materials, including Bi_2_Te_3_-based alloys.^8^ However, the procedure should be done under a carefully controlled inert atmosphere to prevent oxidization of the surface of nanoparticles, because the oxide layer scatters charge carriers significantly, results in poor electrical conduction.

Monolayer doping (MLD) for Si wafers has been proposed by Javey *et al.*^9^ MLD is a method of surface diffusion doping and composed of two steps generally. The first step is adding of precursor molecules having doping elements onto the wafer surface, and the next step is thermal annealing to decompose the precursor molecules followed by diffusing the doping elements into inside of the Si wafer. MLD has been applied to develop various advanced nanostructured devices,^10,11^ including nanowire field effect transistor,^12^ nanoimprinted ultra-shallow junction for three dimensional architecture integrated circuit,^13^ and high-efficiency photovoltaics.^14^ To the best of our knowledge, there is no example of MLD or related surface diffusion doping for Si nanoparticles to synthesize bulk nano-Si for TEs.

Here, we propose an effective method named Surface Diffusion/Sintering Doping (SDSD) to synthesize bulk nano-Si. SDSD is composed of MLD and SPS, in which Si nanoparticles coated with a suitable precursor containing desired amounts of a doping element is sintered by SPS. During the SPS, not only sintering but also diffusing of the doping element from the precursor into Si occur, which realizes to synthesize bulk nano-Si with desired carrier type and concentration.

## 2. Experimental section

Fine Si nanoparticles were prepared by ball-milling from commercial non-doped Si powders (Kojundo Chemical Laboratory Co., Ltd., 3N, particle size: 5 μm). The ball-milling was performed in 2-propanol (Kanto Chemical Co., Inc.) under a nitrogen atmosphere using Microbead mill MSC100 (Nippon Coke & Engineering Co., Ltd.). The size of thus obtained Si nanoparticles was determined by a dynamic light scattering (DLS) method using Microtrac MT3300EXII (MicrotracBEL Co.). In the present study, a phosphonate precursor was selected as the precursor to synthesize P-doped n-type bulk nano-Si. The phosphonate precursor, R-CH_2_-P(=O)(OR_1_)_2_ (R, R_1_ = alkyl), is a proprietary developed material provided by Nitto Denko Corporation. An elemental analysis has proved that the precursor contains 20.1 wt% P. Appropriate amounts of the phosphonate precursor and Si nanoparticles were dispersed in 2-propanol to coat the phosphonate precursor onto the surface of the Si nanopowder. After stirring for 1 hour, the mixtures were centrifuged and decanted to remove the supernatant, then dried in vacuum. The amounts of the phosphonate precursor were set as 0 to 30 wt% in total amounts of the mixtures, corresponding to 0–9% molar parts of precursor phosphorus to Si. The obtained mixtures were consolidating by SPS using SPS-1030 (Sumitomo Coal Mining Co., Ltd.) with uniaxial pressure of 80 MPa at 1373 K for 10 min in an Ar atmosphere to form bulk-state samples.

The phase state was checked at room temperature by powder X-ray diffraction analysis using Ultima IV (Rigaku Co.) with Cu Kα radiation. The microstructure and element distribution of the bulk samples were analyzed by field-emission transmission electron microscopy (FE-TEM) at 200 kV using JEM-2800 (JEOL Ltd.) and energy dispersive X-ray analysis (EDX) using NORAN System 7 (Thermo Fisher Scientific Inc.). *S* and *σ* of the bulk samples were measured simultaneously from room temperature to 1073 K using ZEM-3 (ADVANCE RIKO, Inc.) under a He atmosphere. The Hall coefficient (*R*_H_) was measured by the van der Pauw technique using Resitest8300 (TOYO Co.) from room temperature to 773 K in vacuum under an applied magnetic field of 0.5 T. The Hall carrier concentration (*n*_H_) and Hall mobility (*μ*_H_) were calculated from *R*_H_ assuming a single-band model and a Hall factor of 1; *i.e.*, *n*_H_ = 1/(*eR*_H_) and *μ*_H_ = *σR*_H_, where *e* is the elementary electric charge. *κ* was calculated by *κ* = *αC*_P_*d*, where *α*, *C*_P_, and *d* are the thermal diffusivity, heat capacity, and density, respectively. *α* was measured from room temperature to 1073 K by a flash diffusivity method using LFA-457 (NETZSCH). *C*_P_ was estimated from the Dulong–Petit model, *C*_P_ = 3*nR*, where *n* and *R* are the number of atoms per formula unit and the gas constant, respectively. *d* was calculated from the measured weight and dimensions of the bulk samples. *κ*_lat_ was calculated by subtracting *κ*_el_ from *κ*, where *κ*_el_ was estimated by the Wiedemann–Franz law, *i.e. κ*_el_ = *LσT* (*L* is the Lorenz number: 2.44 × 10^−8^ W Ω K^−2^). Each TE property was repeatedly measured a few times at each measurement temperature; the 10% deviation for *zT* was approximately evaluated from 2%, 3%, and 3% deviations for *S*, *σ*, and *κ*, respectively.

## 3. Results and discussion

**Fig. 1:**
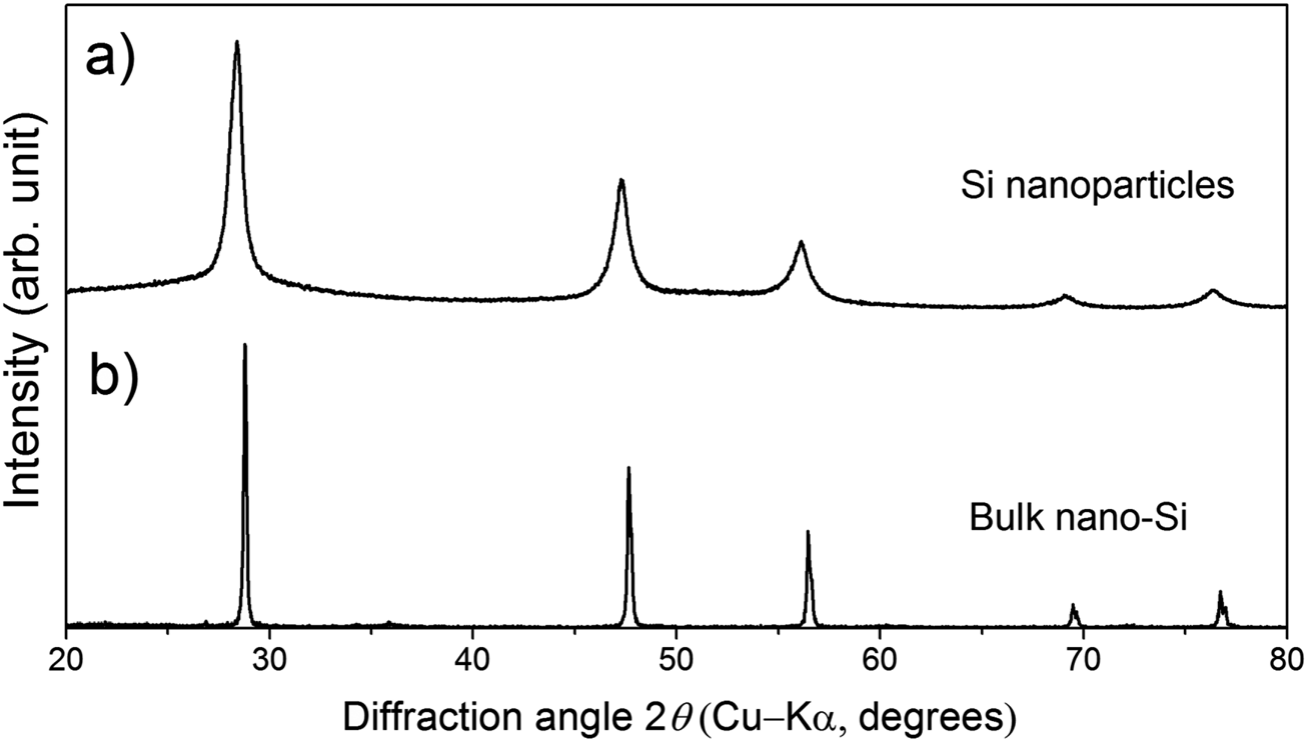
Powder XRD patterns of (a) the Si nanoparticles and (b) a bulk sample whose doping level is 9% molar parts to Si.

The powder XRD pattern of the Si nanoparticles (Fig. 1a) shows broad peaks. The average particle diameter determined by DLS and the crystallite size determined from the XRD pattern are 0.6 μm and 25 nm, respectively. On the other hand, the powder XRD pattern of a bulk sample (Fig. 1b) whose doping level is 9% molar parts to Si shows sharp peaks compared with the Si nanoparticles, meaning that grain growth occurs during SPS.

**Fig. 2:**
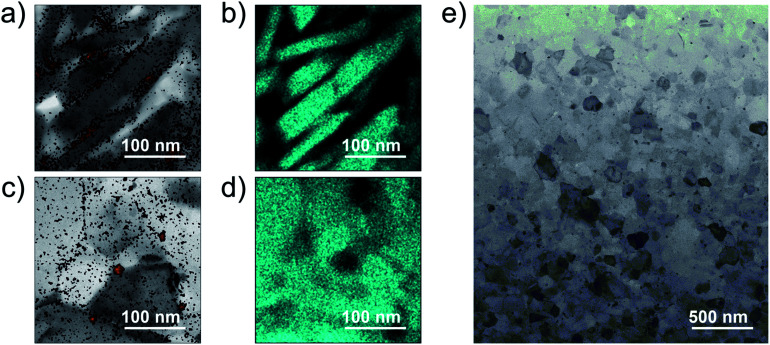
(a and c) TEM image together with the EDX mappings of P and (b and d) EDX mappings of Si. (a and b) For the incompletely sintered sample and (c and d) for the fully sintered sample. (e) STEM image of the fully sintered sample. The doping level of the samples is 2% molar parts to Si.

**Fig. 3:**
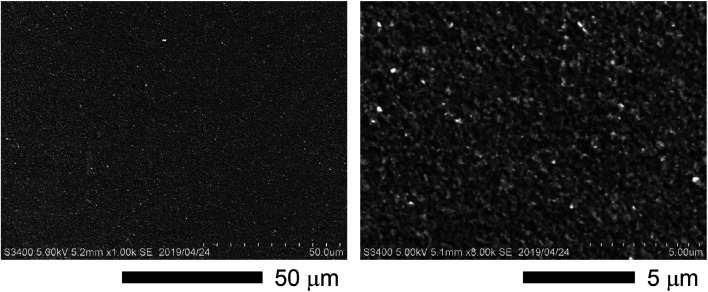
SEM images for the fracture surface of the bulk sample synthesized by SPS at 1373 K. The doping level of the sample is 3% molar parts to Si. The two images are obtained from the same sample; left: low magnification, right: high magnification.

To confirm the surface diffusion, two bulk samples with the same doping level of 2% molar parts to Si were synthesized and characterized by TEM/EDX. One was incompletely sintered by SPS at 873 K and the other was fully sintered by SPS at 1373 K. The density values of the bulk samples synthesized by SPS at 873 K and 1373 K are ∼1.8 g cm^−3^ (∼77% T.D.) and ∼2.3 g cm^−3^ (∼99% T.D.), respectively. The Si grains of the incompletely sintered sample are rectangular-shaped with the size of a few hundred nanometers (Fig. 2a and b). Further, the EDX analysis reveals that almost all P exist at the grain boundaries, meaning that the surface diffusion does not occur at this stage. On the other hand, the fully sintered sample is composed of isotropic grains, in which P is dispersed and almost all P exist in the grains (Fig. 2c and d). As can be confirmed by the TEM image (Fig. 2e), the fully sintered sample shows dense structure with the grain sizes varying from tens to hundred nanometers. The scanning electron microscope (SEM) images for the fracture surface of the bulk sample synthesized by SPS at 1373 K are shown in Fig. 3. The doping level of the sample is 3% molar parts to Si. The SEM observation was performed at room temperature in vacuum using HITACHI S-3400N. From the SEM images, it can be confirmed that a dense and crack-free bulk sample with the grain size of approximately 0.2 μm is obtained. Since the precursor that we used is easy to burn, it is considered that hydrogen and carbon are evaporated in the form of CO_2_ and H_2_O during sintering. According to the quantitative EDX analysis of the sintered bulk samples, the amount of hydrogen and carbon are below the detection limit. These results mean that the surface diffusion as well as sintering doping are successfully carried out during SPS at 1373 K.

**Fig. 4:**
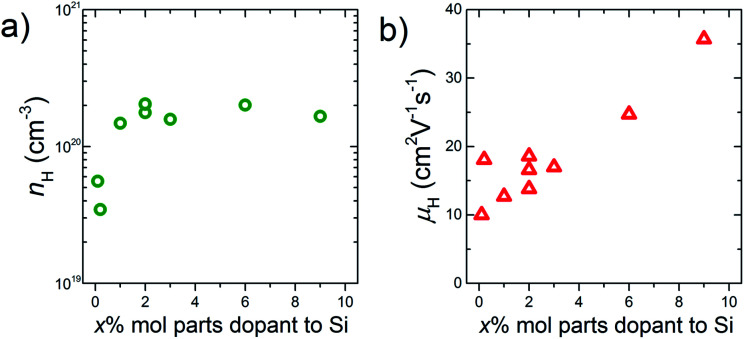
(a) Carrier concentration, *n*_H_ and (b) mobility, *μ*_H_, as a function of the doping level, *x*% molar parts to Si. All the data are obtained at room temperature.

By increasing the amount of the phosphonate precursor added onto the surface of the Si nanoparticles, the *n*_H_ of the bulk samples increases (Fig. 4a). The maximum *n*_H_ value is 2.0 × 10^20^ cm^−3^ obtained at the doping level of 2% molar parts to Si, then saturates. Note that here, the *μ*_H_ continues to increase and keeps high values under the saturated carrier concentration region (Fig. 4b). Native oxide layer on the Si nanoparticles is needed for effective binding of the phosphonate precursor onto the Si surface.^12,15^ When P atoms diffuse from the surface to the inside of the Si nanoparticles during SPS, they may break through the thin oxide layer which results in forming electrically conductive paths.^16^ These conductive paths will contribute to keep high *μ*_H_ even though the oxide layer exists. In the TEM images (Fig. 2), it is confirmed that P infiltrates into the grains by sintering, which provides evidence for the formation of the conductive paths. To obtain more powerful evidence of the formation of the conductive paths, we are planning to perform analyses by means of positron annihilation measurement and impedance measurement.

**Fig. 5:**
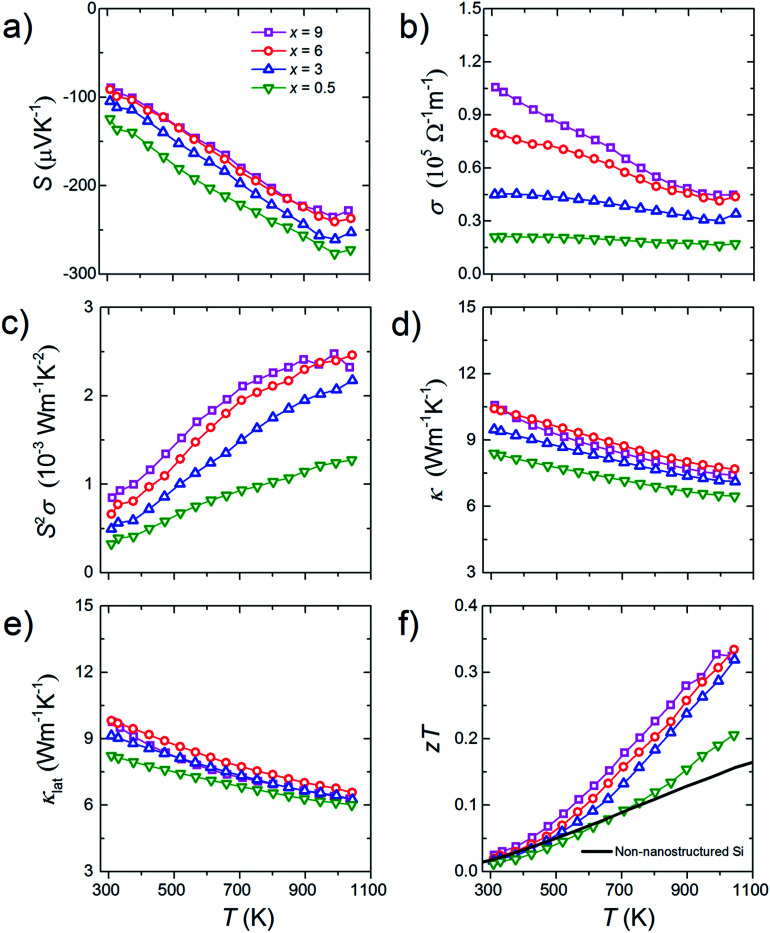
Temperature dependences of (a) Seebeck coefficient, *S*, (b) electrical conductivity, *σ*, (c) power factor, *S*^2^*σ*, (d) thermal conductivity, *κ*, (e) lattice thermal conductivity, *κ*_lat_, and (f) dimensionless figure of merit, *zT* of the bulk samples with the doping level of *x*% molar parts to Si. The *zT* data for typical non-nanostructured n-type bulk Si^17^ are shown as a solid line for comparison.

**Fig. 6:**
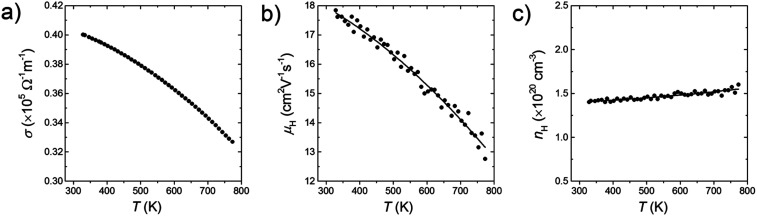
Results of the high-temperature Hall measurement for a bulk sample whose doping level is 3% molar parts to Si. Temperature dependences of (a) electrical conductivity, *σ*, (b) Hall mobility, *μ*_H_, and (c) Hall carrier concentration, *n*_H_. The solid lines are obtained by fitting the data points.

All bulk samples show negative *S* values. Furthermore, for all samples, the absolute *S* show positive temperature dependence while the *σ* show negative temperature dependence (Fig. 5a and b). These results mean that the n-type doping is successfully done. Results of the high-temperature Hall measurement for a bulk sample whose doping level is 3% molar parts to Si are shown in Fig. 6. The *n*_H_ keeps almost constant even though temperature increases, meaning that the carriers are sufficiently doped up to the degenerated level. On the other hand, with increasing temperature, the *μ*_H_ decreases with *μ*_H_ ∝ *T*^−0.2^ (up to 500 K) and *μ*_H_ ∝ *T*^−0.5^ (from 500 to 773 K), suggesting that the scattering mechanism changes at around 500 K. Below 500 K, the impurity scattering (*μ*_H_ ∝ *T*^1.5^) is more predominant than the acoustic phonon scattering (*μ*_H_ ∝ *T*^−1.5^), while above 500 K the acoustic phonon scattering become more predominant. The *S* and *σ* vary in accordance with the doping level, *i.e.*, high doping level leads to low absolute *S* but high *σ*. As the results, high power factor *S*^2^*σ* = 2.5 mW m^−1^ K^−2^ at around 1000 K in maximum is obtained for the bulk sample with the doping level of 9% molar parts to Si corresponding to the carrier concentration of 1.7 × 10^20^ cm^−3^ at room temperature (Fig. 5c). Due to the grain boundary phonon scattering in nanoscale, the *κ* and the *κ*_lat_ are significantly reduced (Fig. 5d and e). The oxide thin layer existed at the grain boundaries may scatter phonons effectively. Owing to the simultaneous realization in the high *S*^2^*σ* and low *κ*_lat_, the enhanced *zT* = 0.34 at around 1000 K in maximum is obtained for the bulk sample with the doping level of 9% molar parts to Si (Fig. 5f). This *zT* value is approximately 2 times larger than typical non-nanostructured n-type bulk Si.^17^

## 4. Conclusion

The present study demonstrates that a proposed method named Surface Diffusion/Sintering Doping (SDSD) is effective to synthesize bulk nano-Si for TEs. P-doped n-type bulk nano-Si synthesized by SDSD shows an ideal properties for TEs, *i.e.*, high carrier concentration (∼2.0 × 10^20^ cm^−3^ at room temperature), high carrier mobility (∼35 cm^2^ V^−1^ s^−1^ at room temperature), and low lattice thermal conductivity (∼6 W m^−1^ K^−1^ at 1073 K) are realized at the same time, which results in the enhanced *zT* of 0.34 at around 1000 K. Since SDSD has high versatility, this method can be applied to other semiconductor/dopant combinations for various applications.

## Conflicts of interest

The authors declare no competing financial interest.

## Supplementary Material
